# The growing potential for multidisciplinary support and technology to enhance person-centred dementia care: a UK and US perspective

**DOI:** 10.3389/frdem.2026.1770741

**Published:** 2026-06-24

**Authors:** Esther V. Loseto-Gerritzen, Ellis C. Dillon, Zlatomira G. Ilchovska, Jen Yates, Richard H. Fortinsky, Mattéa J. Finelli, Michael P. Craven

**Affiliations:** 1 Centre for Dementia, Institute of Mental Health, Mental Health and Clinical Neurosciences, School of Medicine, University of Nottingham, Nottingham, United Kingdom; 2 Center on Aging, University of Connecticut, Farmington, CT, United States; 3 Department of Psychology, University of York, York, United Kingdom; 4 School of Psychology, University of Birmingham, Birmingham, United Kingdom; 5 Centre for Dementia, Translational Medical Sciences Unit, School of Medicine, Biodiscovery Institute, University of Nottingham, Nottingham, United Kingdom; 6 Centre for Dementia, Institute of Mental Health, MindTech HealthTech Research Centre, University of Nottingham, Nottingham, United Kingdom; 7 Human Factors Research Group, Faculty of Engineering, University of Nottingham, Nottingham, United Kingdom

**Keywords:** assistive technology, biopsychosocial model, dementia, disease modifying treatments, multidisciplinary care, person-centred care

## Abstract

Globally more than 55 million people live with dementia, which is estimated to increase threefold by 2050. Dementia poses major challenges for healthcare systems, societies and families, making it a recognised public health priority by the World Health Organization. Advances in pharmacological treatment approaches and new technologies suggest promising improvements in quality-of-life outcomes for people with dementia. However, these new developments may exacerbate existing inequalities in access to dementia treatments and services, depending on how well the organisation and delivery of healthcare services can evolve to successfully link those affected by dementia with treatment options and effective support. In this commentary, we present two important directions to addressing these growing holistic-care challenges and improving life-long quality of life for the patient: firstly, the role of dedicated multidisciplinary support to connect people with dementia to the various services engaged in their care, and secondly the use of technology as an enabler of multidisciplinary care through communication, shared information and coordination within such networks. In particular, we present examples of how artificial intelligence (AI) could be introduced as part of person-centred care during both the diagnosis and monitoring of the disease progression and treatment, to enhance tailoring of personalised therapeutic plans.

## A person-centred biopsychosocial approach

Dementia is associated with a complex range of cognitive, physical, social and emotional challenges that can impact activities of daily living. People with dementia and their families often experience difficulties in accepting and adapting to the diagnosis, alongside disruptions and changes of existing roles, responsibilities and family dynamics. People with dementia can experience a loss of confidence, independence and sense of identity, and a reduction in their social roles and connections (Górska et al., 2018). Family carers report stress and burnout, alongside feelings of isolation, grief and loss (Cross et al., 2018). This affects both the person with dementia and those who support them. Supporting people with dementia and the people around them requires a multidisciplinary team approach that is structured around personhood, person-centred care (Grand et al., 2011; Spector and Orrell, 2010). This complexity of both physical aspects of the condition as well as psychosocial impacts highlights the importance of approaching dementia from a biopsychosocial perspective. The biopsychosocial approach incorporates the psychosocial (e.g., emotional wellbeing, the environment, and social and personal psychology) and biological (e.g., age, overall physical health and genetics) aspects of dementia (Spector and Orrell, 2010). Central to this approach is finding a balance between one’s abilities and disabilities, opportunities and challenges and to tailor support towards individual needs, abilities and preferences throughout the dementia trajectory (McDermott et al., 2019). This is in line with the concept of social health (Huber et al., 2011) and its operationalisation in dementia (Dröes et al., 2017).

## Multidisciplinary care and support

Dementia care relies on multidisciplinary expertise before, during and post-diagnosis. Where in the UK Memory Assessment Services are largely psychiatry-led and delivered through Mental Health Trusts, pre- and post-diagnostic support and therapies still tend to include a range of health and social care providers and specialties including psychiatrists, neurologists, geriatricians, neuropsychologists, nurse practitioners, physical and occupational therapists, nutritionists, social workers, domiciliary care workers, and primary care teams, each of which has access or knowledge of only a piece of the whole puzzle of a person’s life circumstances (Grand et al., 2011; Crooks and Geldmacher, 2004). Partnership working between different specialties is often assumed to be the case by service users and yet in practice it can be challenging to achieve. The concept of holistic, connected care across services and organisations may not be new, but recent developments in treatments and interventions for dementia place a fresh perspective on the vital nature of this element of care delivery.

Multidisciplinary care can address the impact of dementia on a person’s life by providing a better understanding of all challenges and sources of strength in a person’s life. The importance of multidisciplinary interventions to improve quality of life in people affected by dementia was highlighted by a recent systematic review (Luxton et al., 2025), suggesting that efforts and resources should be focused towards the development of multidisciplinary person-centred interventions (Chalfont et al., 2018). However, in practice, many people with dementia and their families experience a lack of accessible multidisciplinary and person-centred care and support services, and research shows that there are many barriers and inequalities in access to such services (Giebel, 2024). Furthermore, the feasibility of implementing such multidisciplinary approaches within a context of over-stretched and under-funded healthcare systems (e.g., the UK), is currently under-researched.

Multidisciplinary care in dementia diagnosis and follow-up is common in the UK, however, the degree of ongoing coordination and continuity across services can be variable. Such a structured, coordinated and multidisciplinary care approach has been recently implemented in the United States and appears promising. Indeed, the Guiding an Improved Dementia Experience (GUIDE) Model was implemented in July 2024 and provides an excellent example of coordinated, multidisciplinary care for people living with dementia. The GUIDE model is a nationwide, voluntary multidisciplinary, coordinated health care payment and service delivery model that aims to improve quality of life for people living with dementia, reduce burden on their family and other unpaid caregivers, and enable people with dementia to remain in their homes and communities (U.S. Centers for Medicare & Medicaid Services). Components of the GUIDE model draw from a dementia care coordination clinical trial called the Care Ecosystems Model that was found to yield positive outcomes related to quality of life for the person with dementia and the caregiver as well as cost savings due to reduced use of health services (Possin et al., 2019).

In line with a person-centred care perspective, as part of the GUIDE model, people with diagnosed dementia and their caregiver (dyad) are assigned a nurse “navigator” who makes an initial home visit to conduct a home safety evaluation and establish an individualized care plan that accounts for goals, preferences, and needs expressed by both members of the dyad. The navigator then helps implement the care plan and refers the dyad to the clinical centre where a physician on the GUIDE team conducts assessments to determine the severity of dementia symptoms, checks on the status of any other medical conditions, and completes a medication review. In addition to nurses and physicians, GUIDE models in larger health systems can involve health professionals from other disciplines such as social work to further help locate and coordinate community-based services for GUIDE participants. Caregiver training, education and support services, including respite care, are available services to the dyad. The nurse navigator is expected to remain the long-term contact for each enrolled dyad, primarily through monthly telephone contacts to check on progress carrying out the personalized care plan. The navigator also can refer the dyad to non-GUIDE paid for services such as transportation and nutrition services which address social determinants of health.

While a national evaluation over the next eight years of this initial test will determine how well the GUIDE program succeeds in meeting its goals at a population level, it is highly likely that many local GUIDE program evaluations will be conducted throughout the test period to help determine GUIDE impacts on person-centered outcomes beyond the scope of the national evaluation. The care pathways for dementia are complex and successful innovation relies on understanding these well and applying treatment with a focus on the person and their specific situation.

## The need for person-centred and multidisciplinary care to deliver disease-modifying therapies

The advent of disease-modifying therapies (e.g., Donanemab, Lecanemab) for mild cognitive impairment due to Alzheimer’s disease (AD) opens up a new level of opportunity to learn how people living with dementia and their families respond to the availability of these pharmacologic treatments. These therapies offer modest reduction in symptoms (Burke et al., 2023; Cummings et al., 2023; Kurkinen, 2023), and are challenging to implement and deliver, carry high potential risks, and involve numerous appointments and monitoring events. The logistics of this typically fall to family or other carers of the individuals receiving the treatments, where caregivers are typically arranging and coordinating medical care, providing transportation, attending appointments, communicating with health care teams, and monitoring side effects, on top of other caregiving responsibilities and personal obligations. All of this requires mentally taxing management of complex information, from different medical specialists, by the carers. Timely flow of information between these specialists in well-coordinated multidisciplinary teams and communication with the person in a patient-centred manner can be crucial for the successful delivery of these new promising but high-risk disease-modifying treatments.

More than 90% of respondents to a recent US survey, carried out by the Alzheimer’s Association to learn about the public’s perspectives on early detection of Alzheimer’s Disease in the era of new treatments, were willing to take a drug to slow the AD process, with more than half being willing even when there was a moderate-high risk of side effects. Considering the frequent infusions and brain scans for side effects, 74% said these potential barriers would not change their minds about taking the medication (Alzheimer's Association, 2025). Studies have highlighted the desire of participants for more individualized information and to hear from others who already began treatment before making their own decision (Parks et al., 2025). Multidisciplinary approaches are likely to be vital in supporting individuals and their caregivers to organize and manage this type of treatment, and to reduce inequalities in access where people with dementia do not have established informal support.

## Technology and AI to support person-centred and multidisciplinary dementia care

Multidisciplinary care poses challenges related to communication, timely coordination of care and support through different professionals, and information sharing, which could in part be addressed by technology. In this context, technology should be understood not as a parallel innovation, but as an enabler of multidisciplinary care. Digital tools can support shared documentation of goals and preferences, exchange updates between carers and professionals, and track changes in symptoms, function or treatment outcomes. People with dementia and carers use already a wide variety of technologies for support throughout the dementia journey from diagnosis to post-diagnostic support access, and to monitor pharmacological treatments (Neal et al., 2025; Saragih et al., 2022; Verma, 2020; Yi et al., 2021).

Technology has the potential for use in screening and early diagnosis at the stage of mild cognitive impairment, such as the digital clock drawing test (Chan et al., 2022). However, there is some debate about the value of population screening (Brunet et al., 2012; Habbal et al., 2025; Hawkes, 2013). In the near future population screening could provide a feasible solution to allocating resources in light of costly clinical testing and therapies. Despite the possibility of disease-modifying effects of the new therapies, long and variable dementia diagnosis timelines could prevent patients from getting an early enough assessment for optimal use of these therapeutic options, calling for a more precise and timely diagnosis for better treatment outcomes. In the UK, Brain Health Clinics provide an excellent example of technology-enabled pathways for assessment and follow-up and have been proposed as one approach to supporting the safe roll-out of disease-modifying treatments (Butters et al., 2025).

Beyond assisting with disease screening, technology has a wide potential in the monitoring of disease progression. AI has emerging potential in dementia care. Recent work suggests possible applications in monitoring systems using sensor, wearable or smartphone data, while current scoping work on generative AI highlights possible roles for conversational agents, voice-based assistants and digital companions that could potentially support cognitive stimulation, orientation, communication and social connection (Chen et al., 2026). A recent scoping review about the potential for AI in supporting the quality of life of people living with dementia shows promise in monitoring systems that use data from various sensors to detect, for example, movements, activity levels or sleep behaviors, and interpret these and their impact on a person’s health (Steijger et al., 2025). In addition to wearable devices, passive remote data collection can draw on in-home or smartphone-based measures to capture changes in routine, mobility, communication patterns or technology use over time. Remote cognitive and functional assessments are also increasingly available, enabling repeated monitoring outside clinic settings and supporting timely review and coordination of care (Shaik et al., 2025; Venkataraman et al., 2024).

Currently technology can be used to streamline care for people with dementia and to improve communication between carers and different members of the multidisciplinary team. For example, applications allow care home staff to record important information about residents, including needs, preferences, delivery of care, and involvement in activities (Pitts et al., 2015). Systems around care management tend to focus on people at advanced stages of dementia (Lorenz et al., 2017), however, these could also be useful for people with mild-to-moderate dementia. Considering that care networks of people with dementia often include both informal and professional carers, technology can usefully support care at earlier stages. Inlife, a platform for informal carers to interact and share care-related updates and progress enabled efficient care coordination and involvement (Dam et al., 2019).

Furthermore, AI could be used to better make sense of complex data sets, such as the combined health states of dyads in relation to available health system and community services. For example, the success of a transition from hospital to community services for a person with dementia could depend on the mobility of their spouse or partner.

Digitalisation of care pathways may have unintended consequences. The COVID-19 pandemic fast-tracked adoption of technology by people with dementia and carers, especially off-the-shelf and low-cost solutions (e.g., free video conferencing platforms) to access care, socialise, or take part in interventions (Barbosa et al., 2024). This improved access to care and provided a lifeline for many but also highlighted existing and new inequalities (Giebel et al., 2021). There is a risk that digital technologies become the default solution for those unable to access in-person services, without considering significant barriers to such technologies. People who lack the resources, infrastructure or digital literacy to use novel or complex solutions may fall further behind. Real-world implementation also depends on interoperability across health and social care systems, workforce capacity and training, information governance, and sustainable funding. Thus, researchers, developers and professionals need to address digital exclusion in technology implementation, so that they continue to support people with dementia and their families in their individual needs, abilities and preferences (Forsyth et al., 2019). Actions on reducing inequalities and on improving community use of such technologies are recommended by Early, Timely and Quality Psychosocial Interventions in Dementia (INTERDEM) Taskforce on Inequalities in Dementia Care whilst its Assistive Technology taskforce stated that more effort should be made to create awareness about technologies and their accessibility (Giebel et al., 2024; Meiland et al., 2017).

## Conclusion

The development of new therapies and technologies brings promise for people with dementia and those who care for and support them. This commentary highlights that, despite these advances, challenges remain in bringing these new therapeutic and technological advances together in a holistic, person-centred and multidisciplinary care pathway for people with dementia. While such new developments can place additional pressure on healthcare systems that already struggle to meet this challenge, they can also offer new opportunities to reshape dementia care pathways in a way that accommodates new developments and improves care. Such an approach has the potential to enhance continuity of care, improve timely and appropriate support and interventions, reduce avoidable crises, such as delayed response to escalating care needs, preventable hospital admissions and poor transitions between services, and ultimately support better outcomes for people living with dementia and their families.

## Data Availability

The original contributions presented in the study are included in the article/supplementary material, further inquiries can be directed to the corresponding author.
